# Phenotypes and Genotypes in Patients with *SMC1A*-Related Developmental and Epileptic Encephalopathy

**DOI:** 10.3390/genes14040852

**Published:** 2023-03-31

**Authors:** Xiuhua L. Bozarth, Jonathan Lopez, He Fang, Jacqueline Lee-Eng, Zhijun Duan, Xinxian Deng

**Affiliations:** 1 Division of Neurology, Seattle Children’s Hospital, University of Washington, Seattle, WA 98105, USA; 2 Department of Laboratory Medicine and Pathology, University of Washington, Seattle, WA 98195, USA; 3 Division of Hematology, University of Washington, Seattle, WA 98195, USA; 4 Institute for Stem Cell and Regenerative Medicine, University of Washington, Seattle, WA 98195, USA

**Keywords:** developmental and epileptic encephalopathy, epilepsy, *SMC1A*, X-chromosome inactivation, escape, *SMC1A*-DEE

## Abstract

The X-linked *SMC1A* gene encodes a core subunit of the cohesin complex that plays a pivotal role in genome organization and gene regulation. Pathogenic variants in *SMC1A* are often dominant-negative and cause Cornelia de Lange syndrome (CdLS) with growth retardation and typical facial features; however, rare *SMC1A* variants cause a developmental and epileptic encephalopathy (DEE) with intractable early-onset epilepsy that is absent in CdLS. Unlike the male-to-female ratio of 1:2 in those with CdLS associated with dominant-negative *SMC1A* variants, *SMC1A*-DEE loss-of-function (LOF) variants are found exclusively in females due to presumed lethality in males. It is unclear how different *SMC1A* variants cause CdLS or DEE. Here, we report on phenotypes and genotypes of three females with DEE and de novo *SMC1A* variants, including a novel splice-site variant. We also summarize 41 known *SMC1A*-DEE variants to characterize common and patient-specific features. Interestingly, compared to 33 LOFs detected throughout the gene, 7/8 non-LOFs are specifically located in the N/C-terminal ATPase head or the central hinge domain, both of which are predicted to affect cohesin assembly, thus mimicking LOFs. Along with the characterization of X-chromosome inactivation (XCI) and *SMC1A* transcription, these variants strongly suggest that a differential SMC1A dosage effect of *SMC1A*-DEE variants is closely associated with the manifestation of DEE phenotypes.

## 1. Introduction

Next-generation sequencing has accelerated the discovery of important genes enriched in pathogenic de novo variants in severe, often previously undiagnosed, developmental disorders [1]. One such gene is the X-linked *SMC1A* (MIM: 300040)*. SMC1A* is an X-linked gene that escapes X-chromosome inactivation (XCI) in females with an estimate of ~30% expression from the allele on the inactive X chromosome (Xi) [2,3,4,5]. *SMC1A* encodes a protein dimerizing with SMC3 to form the core of cohesin, a multisubunit protein complex involved in chromosome segregation, DNA repair, and regulation of chromosomal architecture [6,7]. Pathogenic variants in *SMC1A* as well as other cohesin subunit-encoding and cohesin regulatory protein-encoding genes, can often cause Cornelia de Lange syndrome (CdLS), a developmental condition involving multiple systems. The common features in CdLS include intellectual disability (ID), distinctive facial features with synophrys, short nose, concave nasal ridge, ruptured nasal tip, long and/or smooth philtrum prenatal and postnatal growth retardation, and congenital upper limb anomalies ranging from small hands to severe reduction anomalies of the forearms. Some patients have heart malformations, cleft palate, and nasolacrimal duct obstruction [8]. Multiple genes are associated with CdLS, including heterozygous pathogenic variants in *NIPBL*, *RAD21*, *SMC3*, and *BRD4*, or hemizygous pathogenic variants in *HDAC8 and SMC1A*. *NIPBL* variants are the most common and associated with 60 to 70% of CdLS patients. *SMC1A* pathogenic variants, mostly missense and small in-frame mutations, were found in about 5% of CdLS patients with a male-to-female ratio of 1:2, often associated with mild CdLS phenotypes [9,10]. The *SMC1A* variants in these patients do not affect their overall expression and are protein-sparing, suggesting a dominant negative effect of these mutant SMC1A proteins that could form partially functional cohesin and result in mild CdLS [5,11].

Intriguingly, cases have been increasingly reported of females with heterozygous de novo *SMC1A* pathogenic variants, mostly loss-of-function mutations (LOFs). These variants are associated with a more severe clinical phenotype of developmental and epileptic encephalopathy (DEE), a devastating form of early onset intractable epilepsy beginning in infancy and associated with global developmental delay, cognitive dysfunction, and ongoing epileptiform activity [9,12,13,14,15,16,17,18,19,20,21,22,23,24]. Most reported cases did not show significant brain MRI abnormalities, but cases with holoprosencephaly have been reported [15,23]. Other findings in brain MRI include thin corpus callosum, enlarged extra-axial space, cerebral volume loss, cerebellar atrophy, small cavum septum, and mild periventricular white matter abnormalities [12,13,15]. *SMC1A*-DEE is clearly distinct from *SMC1A*-related CdLS but resembles the clinical phenotype of Rett syndrome (RTT), a severe DEE mostly caused by heterozygous mutations of the X-linked *MECP2* gene, which encodes a chromatin regulator to mediate DNA methylation and gene repression in the brain [9,25]. This suggests a novel link between *SMC1A* and brain development in females.

It is unclear how these *SMC1A* LOF variants cause severe DEE instead of CdLS. Here, we report on the characterization of phenotypes and genotypes of three female patients with de novo pathogenic *SMC1A*-DEE variants, including a previously unreported variant affecting a transcriptional splicing site as well as the XCI patterns and *SMC1A* transcription. We further summarize 41 *SMC1A*-DEE variants reported (including the cases presented here) to characterize common and patient-specific phenotypic features and associated variants for insights related to the etiology of *SMC1A*-DEE.

## 2. Materials and Methods

Patients: Under one approved IRB (IRB #: STUDY00001445), we recruited three family trios with heterozygous *SMC1A*-DEE variants at Seattle Children’s Hospital (SCH, Seattle, WA, USA). Data on clinical phenotypes were collected by chart reviews and evaluations by their neurologists. Whole exome sequencing (WES) was performed for blood samples of all three patient family trios. The most representative transcript, Genbank accession NM_006306, is used when examining the location of these variants on *SMC1A* mRNA. Informed consent was obtained from all affected individuals or their guardians per the specifications of Seattle Children’s Hospital and the University of Washington (Seattle, WA, USA), including consent to publish photographs.

Genomic DNA isolation and confirmation of *SMC1A* variants: Genomic DNA (gDNA) was extracted from whole blood from patient families using the Qiagen DNeasy Blood & Tissue Kit (Qiagen, Valencia, CA, USA). gDNA was subject to PCR with primer pairs flanking the *SMC1A* variants, followed by Sanger sequencing for confirmation. The primer information is in Table S1. Note that the blood sample of the father of patient 3 (P3) was not available. 

RNA isolation and expression analysis: Total RNA was extracted from whole blood using the QIAamp RNA Blood Mini Kit (Qiagen, Valencia, CA, USA) with on-column DNase I digestion. cDNA was produced from reverse transcription (RT) of total RNA and used for PCR with specific primers flanking the variants to detect *SMC1A* transcripts. Sanger sequencing was then performed to confirm alternative transcripts in patient 1 (P1) carrying a splice-site variant or the missense transcript in patient 3. Note that to confirm the transcript with exons 4–5 skipping in P1, pre-amplified PCR product from cDNA with primers flanking exons 2–6 was subjected to HindIII digestion that only cut the wild-type transcript carrying a HindIII site in exon 5. The digested product was diluted and used for another round of PCR to only amplify the transcript with exons 4–5 skipping followed by Sanger sequencing. The primer information is in Table S1.

XCI evaluation: Methyl-sensitive PCR-based assay for the *AR* (androgen receptor) locus (i.e., HUMARA assay) containing the polymorphic trinucleotide repeats was performed for gDNA from blood as previously described with minor modifications [26]. In brief, DNA samples were PCR amplified for the CAG repeat region (CTG from the minus strand) in the *AR* exon 1. In parallel, DNA samples were digested with the methyl-sensitive restriction enzyme HpaII, followed by PCR. Sanger sequencing and comparison of the amplified product from DNA with and without digestion were used to determine the presence of polymorphic repeat in test samples and the extent of XCI skewing. For patient 2 (P2), showing no polymorphic CAG repeats of *AR* (i.e., both paternal and maternal alleles have the same number of repeats), a similar methyl-sensitive PCR-based assay was performed for the *RP2* (retinitis pigmentosa 2) locus containing the polymorphic CAAA repeats [27]. The primer information is in Table S1.

## 3. Results

Clinical phenotypes (Table 1 and Table 2): P1 is currently an 11-years old Caucasian girl. She was born at full term. At birth, she weighed 2.35 kg (1.7th centile), her length was 45.7 cm (3rd centile), and her head circumference (OFC) was 32.5 cm (12th centile). She had mild neonatal jaundice that did not require treatment. She does not have facial CdLS features. She has very mild 2–3 syndactyly on both feet, severe pes planovalgus deformity, the myopia of both eyes with astigmatism, diffuse hypotonia, and mild ptosis bilaterally. She had G-tube placement at the age of 7 years. She had a history of normal development until approximately 4 months of age when she had her first seizure. At the age of 11 years, she has a profound global developmental delay. She remains nonverbal. She is able to grab objects with both hands and crawl but cannot stand or walk independently. Her weight is at the 10th centile, her height is at the 15th centile, and her OFC is at the 1.7th centile. Her seizures started at the age of 4 months. She has multiple seizure types, and seizures are refractory to treatment. Her seizure types include generalized tonic–clonic, myoclonic, atonic, atypical absence seizures, and focal onset motor and nonmotor seizures. She has frequent clusters of seizures and status epilepticus, requiring hospitalization. She also has vagal nerve stimulation placement and a trial of modified Atkin’s diet that did not show clear efficacy. She continues to have frequent seizures and is on four AEDs, including topiramate, perampanel, epidiolex, and lacosamide.

P2 is currently an 8-years old Hispanic girl. She was born full term. At birth, she weighed 3.26 kg (55th centile), her length was 48.6 cm (23rd centile), and her OFC was 33.1 cm (14th centile). She was found to have severe axial and appendicular hypotonia, neonatal hyperbilirubinemia, and feeding difficulties. She has a wide cleft palate, microform cleft lip, synophrys of her eyebrows, tapered fingers, and hirsutism. She had G-tube placement at 9 months of age. She had a heart murmur, and her echocardiogram revealed left ventricular hypertrophy at the age of 9 months. Her kidney ultrasound revealed right hydronephrosis and small bilateral cysts. She also has hyperopia and astigmatism at the age of 15 months on ophthalmological examination. She has mild cortical visual impairment on visual evoked potential test at the age of 20 months. She was found to have sleep apnea. She lacked progression of developmental milestones. At the age of 8 years, she has a profound global developmental delay. She remains nonverbal and does not have good head control. There is a paucity of spontaneous movements. Her current weight is at the 43rd centile, height is at the 0.2th centile, and OFC is <1st centile. Her seizures started at the age of 2 months, and developmental infantile spasms at the age of 9 months. Her seizure types include tonic/atonic, tonic–clonic, focal motor, and focal nonmotor seizures. She is still on two AEDs, including clobazam and levetiracetam. A modified Atkin’s diet was partially effective.

P3 is currently a 9-years old Asian descendant girl. She was born at 36 + 6/7 weeks of gestational age. At birth, she weighed 2.2 kg (<1st centile), her length was 44.5 cm (<1st centile), and her OFC was 30.5 cm (<1st centile). She has mild synophrys, a mid-low anterior hairline, a mild small jaw, small hands and feet, shortened fifth digits, and mild proximal placement of her thumbs. She has myopia, astigmatism, and mild bilateral ptosis. She had a history of gastroesophageal reflux disease as an infant. Her audiologic testing showed a mild conductive hearing loss, and BAER revealed a mild conductive high-frequency hearing loss at the age of 1 year. Her renal ultrasound study at the age of 1 year revealed bilateral minimal central pelviectasis. At the age of 9 years, she has a severe global developmental delay. She is a minimally verbal communicator who uses vocalizations, occasional word approximations, signs, gestures, and picture symbols to communicate. She is able to understand picture symbols representing common actions, objects, and people, as well as some descriptive words. She uses a combination of methods, including vocalizations, word approximations, sign language, and picture symbols, on a speech device to make a variety of requests and communicate her basic needs. She is able to walk and run independently with a mild wide-based gait. Her current weight is at the 83rd centile, height at the 71st centile, and OFC at the 10th centile. She had a history of a cluster of febrile seizures at the age of 2 years and 11 months. Her unprovoked seizures started at 3.5 years old. Her seizure types include tonic–clonic, tonic/atonic, and myoclonic. Her seizures cluster and occur from less than a month up to seven seizures per week. She did not respond to Atkin’s diet. She currently is on two AEDs, including clobazam and brivaracetam. Brivaracetam significantly reduced her seizure burden.

Diagnostic workup results: *SMC1A* mutations in all three patients were originally identified by WES as the only pathogenic variants linked to DEE (Table 3). WES for P1 showed heterozygous de novo likely pathogenic novel variant in the X-linked *SMC1A* gene at c.615+5G>A in intron 4. P1 underwent multiple genetic tests without detected abnormalities, including oligonucleotide array, single gene sequencing for *SCN1A*, *CDKL5*, *SLC2A1*, sequencing, deletion, and duplication testing for *MECP2*, and the epiSEEK panel (Courtagen, Woburn, MA, USA) that included 71 known epilepsy genes. She has had normal metabolic screenings for plasma amino acids, urine organic acids, acylcarnitine profile, transferrin isoelectric focusing to screen for congenital disorders of glycosylation, AASA, and P6C levels to screen for pyridoxine-dependent epilepsy.

WES for P2 showed a heterozygous de novo pathogenic indel variant in *SMC1A* at c.1636_1638del ATT. P2 had normal chromosomal microarray analysis prior to WES. She has had normal metabolic screenings for plasma amino acids, urine organic acids, acylcarnitine profile, transferrin isoelectric focusing to screen for congenital disorders of glycosylation, and urine quantitative mucopolysaccharides.

WES for P3 showed a heterozygous de novo pathogenic missense variant in *SMC1A* at c.1487G>A (p.R496H). P3 had normal genetic testing prior to WES, including prenatal karyotype and chromosome microarray analysis on chorionic villi sampling and postnatal *NIPBL* sequencing. She has had normal metabolic screenings for ammonia, lactate, pyruvate, amino acids, acylcarnitine profile, urine oligosaccharides, and urine organic acids.

Brain MRI in P1 showed a small cyst in the left anterior temporal region at the age of 7 months and at the age of 9 years with no change. Brain MRI in P2 at the age of 2 years was normal. P3 did not have a brain MRI.

EEG showed diffuse background slowing in all three patients (Table 2). P2 had a history of hypsarrhythmia on her EEG. Her EEG did not have a posterior dominant rhythm and did not have normal sleep architecture at the age of 3 years.

**Figure 1:**
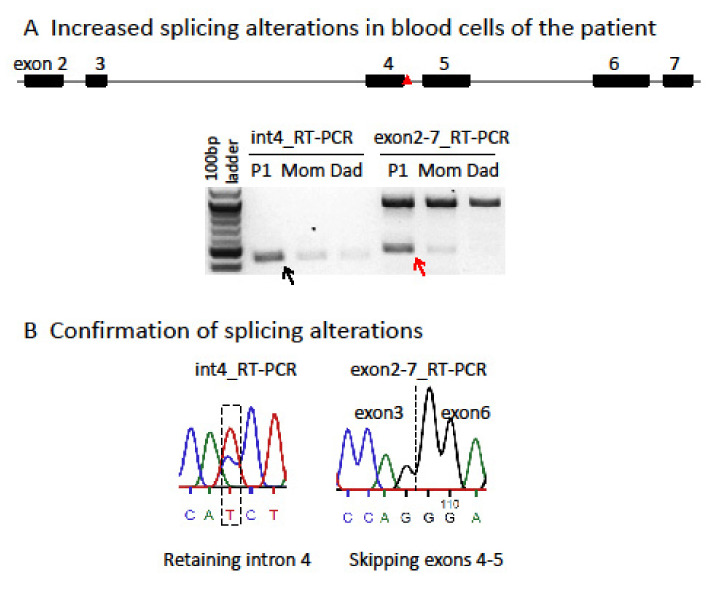
The SMC1A splice-site variant in P1 impacts transcriptional splicing. (**A**) c.615+5G>A (red triangle) in intron 4 strongly increases two alternative splicing events in P1, retaining intron 4 (black arrow; 438 bp) and skipping exons 4–5 (red arrow; 479 bp), as shown by RT-PCR using primer pairs specific for intron 4 transcript or spanning exons 2–7 and RNA from whole blood of the P1 family trio. The large band for the wild-type transcript containing exons 4–5 detected by primer pairs spanning exons 2–7 is 922 bp. (**B**) Sanger sequencing of RT-PCR products confirms these two alternative splicing events. Both abnormally spliced transcripts cause frameshifts and premature translational stop codons and thus cannot produce functional proteins, consistent with loss of function (see Table S2).

**Figure 2:**
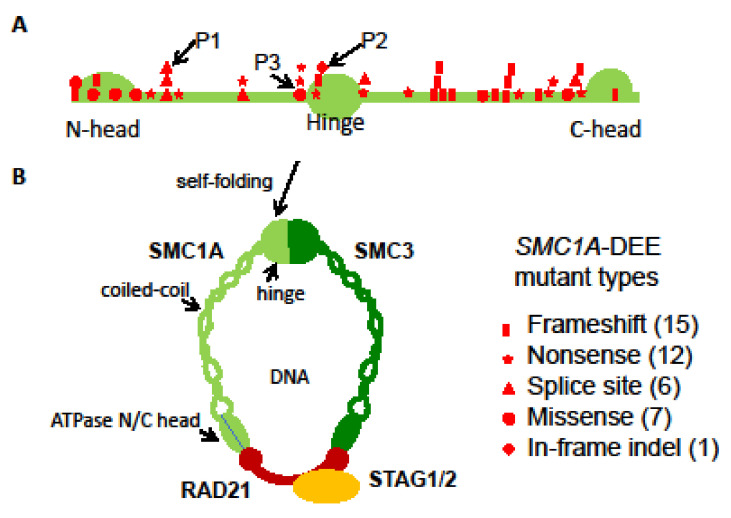
*SMC1A*-DEE variants. (**A**) Location and types of 41 *SMC1A*-DEE variants, including 39 reported and 2 new ones shown on the SMC1A protomer (see Table 1). Variants of our patients 1–3 are marked. (**B**) After self-folding of the coiled-coil, SMC1A and SMC3 form a hinge domain at one end and bind to RAD21 at the other end (N/C head). Finally, RAD21 interacts with STAG1/2 and forms a ring-shaped cohesin complex to fold chromatin. The number of assembled cohesin would be reduced by these LOFs.

**Figure 3:**
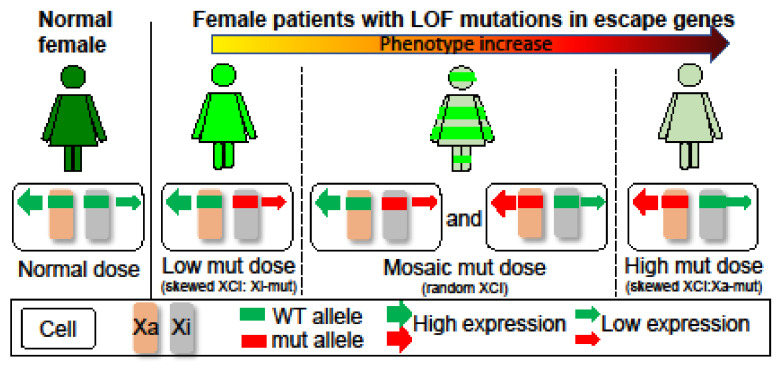
Impact of XCI on X-linked disorders due to heterozygous LOF mutations in escape genes. Normal females have a dose of products from wild-type (WT) alleles on the Xa and Xi. Patients with skewed XCI have a differential dose reduction depending on which X carries the mutation (mut). This would lead to a mild phenotype in patients with Xi-mut, whereas a most severe phenotype in patients with Xa-mut. Thus, skewed XCI towards the mutant allele could alleviate the phenotype. In contrast, patients with random XCI have a mosaic reduction due to mixtures of cells expressing Xa- or Xi-mut, leading to a phenotype between patients with Xi-mut or Xa-mut. In addition, XCI mosaicism could cause cellular interference at tissue levels and lead to a different phenotype, as is the case for *PCDH19* clustering epilepsy.

Characterization and comparison of de novo *SMC1A-DEE variants* in our study and literature: Sanger sequencing for PCR products flanking the variant confirmed that P1 carries an unreported de novo heterozygous splice-site mutation (c.615+5G>A) in intron 4 of *SMC1A* (Figure S1). Interestingly, two variants (c.615G>A and c.615+1G>C) were recently reported to affect the same splice site (Table 3) [15,19]. Note that in the report by Symonds et al. [15], case 3 is identified as a splice-site variant at c.549G>A, which is at the exon junction of the *SMC1A* transcript NM:001281463 but not NM_006306. Thus, it corresponds to c.615G>A in NM_006306. This variant has also been reported in a new patient (case 12) [19]. In addition, two other variants (c.616-2A>G, VCV000159962.5; c.615+2T>C, VCV000804008.1) out of a total of seven pathogenic splicing variants deposited in ClinVar also affect this splice-site but has not been reported in any literature, strongly indicating a pathogenic splicing hotspot. RT-PCR for RNA from blood using primers specific for intron 4 or spanning exons 2–7 showed two alternative transcripts in P1, which were not or only barely detected in the mother or father (Figure 1). Using a different pair of primers spanning exons 2–6 showed a similar result. Sanger sequencing confirmed these alternative transcripts. This is consistent with the prediction of splice-site interference from this variant. Both abnormally spliced transcripts cause frameshifts and premature translational stop codons and thus cannot produce functional proteins consistent with LOF (Table S2 and Figure 2). We performed a methyl-sensitive PCR-based assay for XCI evaluation and found that P1 has a random XCI pattern in blood, i.e., an equal chance of the maternal or paternal X being the Xi (Figure S2). Given that *SMC1A* escapes XCI with ~30% of expression levels from the allele on the Xi [5], random XCI in P1 would lead to a mosaic dosage effect at the tissue level (Figure 3), i.e., expression levels of this variant are ~two-fold lower in cells when it is located on the Xi versus in cells when it is located on the active X chromosome (Xa).

A de novo heterozygous in-frame indel (c.1636-1638delATT) was previously reported in P2 by Seattle Children’s Hospital in 2016 (Table 3) [21] and confirmed by Sanger sequencing (Figure S1). This variant results in missing an isoleucine residue at position 546 (p.Ile546del) in the hinge. This residue is highly conserved across species and is important for hinge domain dimerization with SMC3 (Figure 2) [9,28,29]. We hypothesize that this variant affects the assembly of the cohesin complex and thus has a similar dosage effect as those LOF ones (Figure 2). P2 also has a random XCI pattern in blood, again suggesting a mosaic dosage effect (Figure 3 and Figure S3).

P3 is a previously unreported patient carrying a de novo missense variant (c.1487G>A; Table 3), which has been reported in three other unrelated female probands previously classified as mild CdLS cases with considerable growth and development variability [28]. Sanger sequencing confirmed this variant in P3 but not in the mom. This mutation leads to an arginine to histidine substitution (p. R496H) at the junction between the coiled coil and the hinge (Figure 2). This region is also highly conserved and predicted to be vital for hinge domain dimerization [28]. Thus, similar to the variant in P2, this missense variant could have a dosage effect as those LOFs. Interestingly, seizures have also been noticed in these patients, including our P3. P3 had an increased frequency of seizures starting at the age of 3 years but not as severe as in P1 and P2 (Table 1). XCI testing showed a completely skewed inactivation of the paternal X in P3 blood (Figure S4), allowing examination of the *SMC1A* escape status. RT-PCR using cDNA-specific primers flanking this variant followed by Sanger sequencing reveals expression of both wild-type (WT) and variant alleles in blood cells with skewed XCI, consistent with the escape of *SMC1A* from XCI (Figure S5). In addition, the peak ratio between the WT allele (i.e., G) and the variant (A) is increased by ~4-fold from the cDNA-derived PCR product compared to that from the gDNA-derived PCR product, suggesting most of *SMC1A* expression is from the WT allele. Given that the allele on the Xa is expressed at a higher level than that on the Xi, we can infer that this variant is on the paternal Xi, and the expression level of the variant (i.e., the escape level) is about 25% of that of the WT allele on the maternal Xa. This suggests a mild dosage effect and is consistent with an alleviated DEE phenotype in P3 (Figure 3). 

Our characterization of these three heterozygous *SMC1A*-DEE variants and their XCI patterns suggests a possible link between *SMC1A* dosage reduction to the severity of phenotype (Figure 3). To further test this, we surveyed 41 known *SMC1A*-DEE variants, including ours and one unreported, and characterized common and patient-specific features (Table 3) [9,12,13,14,15,16,17,18,19,20,21,22,23,24]. Clearly, *SMC1A*-DEE variants are mostly LOF caused by nonsense, frameshift, or splice-site interference (Figure 2). Seven of the eight non-LOF variants, including two from our cohort (variants in P2 and P3) and one unreported (c.3241A>T) for which the patient will be included in our cohort in the future, are located in the N/C-terminal ATPase head or in the central hinge domain. These regions are predicted to disrupt interactions with SMC3 or RAD21, respectively, and to prevent cohesin assembly, thus probably mimicking LOF (Figure 2) [9,28,29]. All 41 variants occurred in heterozygous girls, suggesting lethality in males. These variants result in intractable early-onset epilepsy (with some features similar to *PCDH19* clustering epilepsy), progressive neurodevelopmental impairment, and (in some) regression. Six of these *SMC1A* LOFs have even been linked to the most severe midline brain defect, holoprosencephaly (HPE) [23]. Interestingly, two of the HPE variants were also reported in unrelated patients with a minor phenotype, including late-onset DEE and no midline brain defects [17,24]. Overall, ten variants are associated with patients, including our P3, who show a lesser phenotype: seizure onset between birth to 15 months with variable response to antiseizure medication and less severe GDD with independent walking and limited expressive speech. This is consistent with the study by Chinen et al. [24], indicating that the age of seizure onset at 15 months or later in six cases is associated with better motor development. When the XCI status is available, these patients with less severe DEE are more often with skewed XCI. Overall, these suggest that different XCI status probably contributes to a patient-specific manifestation of the disease as proposed by our dosage model (Figure 3).

## 4. Discussion

The phenotype of *SMC1A*-DEE is clearly distinct from CdLS but resembles *MECP2*-linked RTT: early onset intractable epilepsy and severe or profound intellectual disability. In addition, heterozygous variants of other chromatin regulators (e.g., HDAC8, SATB2, and HNRNPU) also often cause RTT-like phenotypes [25]. These clinical and genetic observations strongly suggest a dosage-sensitive role of chromatin components in early brain development. Considering that RTT-like phenotypes occur in the early postnatal period, mutations of these master chromatin regulators could disrupt multiple pathways critical for the early development of complicated neuronal networks, such as synapse formation that peaks sometime in the first year of life and is the basis of much of the learning [30]. Indeed, it has been reported that in mice heterozygous for a knockout of SMC3, which encodes the SMC1A partner and the core subunit of cohesin, altered neural gene expression during cortical synapse formation leads to defective synapse development and anxiety-related behavior [31].

The etiology of *SMC1A*-DEE has yet to be studied due to limited patient numbers and the inaccessibility of brain tissue. A phenotype and genotype comparison of *SMC1A*-DEE patients in our study suggests an X-specific differential dosage effect that is influenced by the XCI status (Figure 3) [32]. Differential SMC1A insufficiency in *SMC1A*-DEE individuals with random XCI could further lead to cellular interference (i.e., interference between cells with differential levels of SMC1A) at tissue levels, similar to the proposed for the etiology of *PCDH19* clustering epilepsy [33]. Interestingly, *SMC1A*-DEE and *PCDH19* clustering epilepsy share some epilepsy-related features, e.g., drug resistance and seizure clustering [15]. In contrast, *SMC1A*-DEE individuals with skewed XCI toward the LOF variant, such as our P3, would have a minor deficiency compared to that in normal females with expression from both alleles and thus show a less severe phenotype (Figure 3). Additional products from an expression of the allele of XCI-escape genes on the Xi have been proposed to underlie sex differences between males and females, including brain development, and contribute to phenotypes associated with X aneuploidy [32,34]. Interestingly, the ortholog of *SMC1A* in mice does not escape XCI in the brain [35], and thus, studies of *SMC1A*-DEE in mouse animal models could fail to recapitulate the differential dosage effect of SMC1A in humans. In vitro neural models using patient-derived induced pluripotent stem cells carrying the *SMC1A*-DEE variant on the Xi or Xa could be more applicable in functional studies of *SMC1A*-DEE. Our collections of blood cells from rare *SMC1A*-DEE patients will be valuable for such studies.

## 5. Conclusions

Characterization of *SMC1A*-DEE variants as LOFs suggests a dosage-sensitive role of *SMC1A* in early brain function and development. The XCI status in female *SMC1A*-DEE patients plays an important role in the manifestation of DEE phenotypes.

## 6. Future Directions

There is no effective treatment for this patient population. Understanding the disease mechanism has the potential to provide new therapeutic targets. In vitro neural models using patient-derived induced pluripotent cells could be an important tool to understand the pathology, while animal models will provide further in vivo insights for this condition. Both disease models could provide new revenues for personalized therapeutic treatments. Additionally, understanding the natural history of *SMC1A*-DEE will lay the important groundwork for clinical trial readiness by defining disease severity and change in the symptoms over time. It is especially crucial for disease-modifying treatment, such as gene therapy for increasing *SMC1A* expression. Thus, multicenter studies and international collaboration are important steps toward understanding this rare genetic disorder and uncovering new therapies.

## Figures and Tables

**Table 1:** Patients’ general characteristics.

	Patient 1	Patient 2	Patient 3
Full term	Yes	Yes	No, 36 + 6/7 wk
Congenital microcephaly	No	No	Yes
Postnatal microcephaly	Yes	Yes	Yes initially
Hypotonia	Yes	Yes	Yes
Expressive language	None	None	A few words
Encephalopathy	Profound	Profound	Severe
G-tube	Yes (4 yr)	Yes (9 M)	No
CDLS features	No	Minor	Minor
Independent walking	No	No	Yes
Precocious puberty	Yes	No	No
Cardiac abnormalities	No	Left ventricular cardiomyopathy	No
MRI:	A small cyst in left anterior temporal region (age 7 M and 9 yr)	Normal (2 yr)	No

M = month; yr = years; wk = weeks.

**Table 2:** Patients’ characteristics in epilepsy.

	Patient 1	Patient 2	Patient 3
Age of seizure onset (M)	4	2	35
Intractable epilepsy	Yes	Yes	Yes
Epilepsy syndrome			
LGS	Yes	No	Yes
Epileptic spasms	No	Yes (9 M)	No
History of status epilepticus	Yes	Yes	No
Seizure burden	Weekly and up to daily	Weekly and up to daily	Less than monthly
Seizure Types			
Tonic/atonic	Yes	Yes	Yes
Tonic clonic	Yes	Yes	Yes
Myoclonic	Yes	No	Yes
Focal onset, motor	Yes	Yes	No
Focal onset, nonmotor	Yes	Yes	No
Atypcial absence	Yes	No	No
Cluster seizures	Yes	Yes	Yes
Age starting AED	4 M	2M	47M
Current AED:	Topiramate, Perampanel, Epidiolex, Lacosamide	Clobazam Levetiracetam	Brivaracetam Rufinamide Clobazam
Past AED	Zonisamide, Valproic acid, Clobazam Oxcarbazepine, Levetiracetam, Rufinamide, Lamotrigene, Brivaracetam	Phenobarbital, Oxcarbazepine, ACTH, Viagabatrin, Diazepam, Zonisamide	Valproci acid, Levetiracetam
AEDs: severe adverse effects	No	No	No
Ketogenic/modified Atkin’s diet	Yes, no efficacy	Yes, partial efficacy	Yes, no efficacy
Vagal nerve stimulator	Yes, unclear efficacy	No	No
Epilepsy resection surgery	No	No	No
EEG			
Normal Yes/No	No	No	No
Posterior dominant rhythm	Yes, slow for age	No	Yes, slow for age
EEG background	Diffuse slow	Diffuse slow, history of hypsarrhythmia	Diffuse slow
Sleep architecture	No	No	Yes
Interictal pattern	Generalized; multifocal, more posteriorly	Multifocal	Generalized spike wave
GPFA	Yes	No	No
Slow Spike wave	No	No	Yes

AED = antiseizure medication; PDR = posterior dominant rhythm; Ruf = rufinamide; GPFA = Generalized paroxysmal fast activity; M = months.

**Table 3:** Information of 41 SMC1A-DEE variants.

Variant #	Nucleotide Changes	Amino Acid Changes	Domain	Type	XCI	Ref.	Age of Sz Onset	Speech	ID	Walking	MRI
1	C.20_23del	Ile7Argfs*42	P1 head	Frameshift	Moderately skewed 81:19	Barañano, 2022 [19] (P6)	15 M	none	yes	No	n/a
2	c.31A>T	Asn11Tyr	P1 head	Missense	n/a	Huisman, 2017 [9] (P1)	2.5 M	n/a	yes		normal
3	c.140T>G	Phe47Cys	P1 head	Missense	Random 74:26	Barañano, 2022 [19] (P3)	3 M	none	yes	No	n/a
4	c.157dup	Thr53AsnfsX34	P1 head	Frameshift	n/a	Huisman, 2017 [9] (P2)	5 M	n/a	yes	n/a	cerebral volume loss
5	c.287G>C	Arg96Pro	P1 head	Missense	Random	Barañano, 2022 [19] (P11)	18 M	n/a	yes	n/a	n/a
6	c.421G>A	Glu141Lys	P1 head	Missense	highly Skewed 100:0	Barañano, 2022 [19] (P1)	2.5 M	none	yes	n/a	n/a
7	c.511C>T	Arg171Ter	Coil	Nonsense	Random	Symonds, 2017 [15] (P7)	4 Weeks	none	yes	No	normal
8	c.615G>A	Glu205fs*	Coil	Splice-site	N/A	(NM:00128146:c.549G>A) in Symonds, 2017 [15] (P3); P12 in Barañano, 2022 [19]	P3 Symonds-4 M; P12 Barañano-13 M	none	yes	No	P3 Symonds-small hemorrhage along posterior falx and tentorium; P12-Barañano-n/a
9	c.615G+1G>C	Glu205fs*	Coil	Splice-site	Moderately skewed 83:17	Barañano, 2022 [19] (P9)	1 M	n/a	yes	No	n/a
10 (P1 in our cohort)	c.615+5G>A	Glu205fs*	Coil	Splice-site	Random	Our cohort P1	4 M	none	yes	no	normal
11	c.694G>T	Glu232Ter	Coil	Nonsense	n/a	Huisman, 2017 [9] (P7)	4 M	n/a	yes	n/a	n/a
12	c.1113+1G>A	Gln371fs*	Coil	Splice-site	n/a	Gorman 2017 [16] (P2)	7 Weeks	none	yes	n/a	n/a
13	c.1114delG	Val372Ter	Coil	Nonsense	n/a	Gorman 2017 [16] (P1)	7 Weeks	none	yes	no	n/a
14 (P3 in our cohot)	c.1487G>A	Arg496His	Close to hinge	Missense	Skewed in our patient P3; N/A in 5 patients from Deardorff	Our cohort P3; Deardorff, 2007 [28] (P3)	35 M	limited	yes	Yes	n/a
15	c.1489C>T	Arg497Ter	Close to hinge	Nonsense	n/a	Fang, 2021 [18]	4 M	none	yes	No	normal
16	c.1495C>T	Arg499Ter	Close to hinge	Nonsense	n/a	Chinen, 2019 [24]; Kruszka, 2019 [23] (P8)	40 M-Chinen, 2019; N/A-	none	yes	Chinen-Yes	ChinenP8- microform of HPE, Kruszka P8-Triventricular ectasia
17	c.1591C>T	Gln531Ter	Hinge	Nonsense	Moderately skewed	Symonds, 2017 [15] (P1)	15 M	none	yes	Yes	normal
18	c.1609delG	Val537Phefs*42	Hinge	Frameshift	highly skewed	Barañano, 2022 [19] (P8)	5 M	none	yes	No	n/a
19 (P2 in our cohort)	c.1636_1638delATT	Ilu546del	Hinge	In-frame	Random	reported in Wenger, 2016 [21]	2 M	none	yes	No	normal
20	c.1900C>T	Gln634Ter	Close to hinge	Nonsense	Moderately skewed 86:14, Mosaic	Barañano, 2022 [19] (P2)	3 M	n/a	yes	n/a	n/a
21	c.1911+1G>T	Thr638Valfs*48	Close to hinge	Splice-site	n/a	Lebrun, 2015 [14]	Neonate	none	yes	no	small frontal lobe, thin CC
22	c.2197G>T	Glu733Ter	Coil	Nonsense	Random	Symonds, 2017 [15] (P4)	5 M	none	yes	No	volume loss
23	c.2364del	Asn788Lysfs*10	Coil	Frameshift	Random	Jansen, 2016 [16] (P1)	9 M	none	yes	No	slightly enlarged ventricles, hypotrophy cerebellar vermis
24	c.2394delA	Lys798Asnfs*31	Coil	Frameshift	n/a	Kruszka, 2019 [23] (P9)	n/a	n/a	yes		semi-lobar HPE
25	c.2394dupA	Arg799fs	Coil	Frameshift	n/a	Barañano, 2022 [19] (P5)	4 M	none	yes	No	n/a
26	c.2421_2562del	Leu808Arg-fs*6	Coil	Frameshift	Moderately skewed 85:15	Jansen, 2016 [16] (P2)	2 M	none	yes	None	mild periventricular white matter abnormalities
27	c.2477delA	p825fs	Coil	Frameshift	n/a	Symonds, 2017 [15] (P8, P9)	28 M in P8; <1 M in P9	P8-none; P9-none	yes	P8-yes	P8-normal; P9-hemi-lobar HPE
28	c.2683C>G	Arg895Gly	Coil	Missense	Skewed-P1; n/a-P2, P9 from Kruszka	Oguni, 2019 [17]; Kruszka, 2019 [23] (P9)	25 M-P1 Oguni, P2 (mother of P1)-Oguni- 12y; n/a- P8-Kruszka	Oguin-none; Kruszka-n/a	P1-yes; P2-normal until sz onset	Oguni-P1-no, P2-yes, Kruszka-n/a	P2: cerebellar atrophy; P9-Kruszka-semi-lobar HPE
29	c.2769dupC	Ser924Glnfs*2	Coil	Frameshift	n/a	Barañano, 2022 [19] (P10)	24 M	no	yes	n/a	n/a
30	c.2834delG	Gly945Alafs*19	Coil	Frameshift	n/a	Kruszka, 2019 [23] (P11)	n/a	n/a	yes	n/a	semi-lobar HPE
31	c.2853_2856delTCAG	Ser951Argfs*12	Coil	Frameshift	Skewed	Goldstein, 2015 [12] (P1)	4 M	n/a	severe	no	mild ventriculomegaly, mildly rotated hippocampal heads
32	c.2873delA	Gln958Argfs*6	Coil	Frameshift	Random	Barañano, 2022 [19] (P7)	3 M	none	Severe	no	n/a
33	c.2923C>T	Arg975Ter	Coil	Nonsense	N/A	Symonds, 2017 [15] (P6)	5 M	Lost speech after status	Moderate to severe	yes	normal
34	c.3046_3048delGTGinsG	Val1016Alafs*28	Coil	Frameshift	Random	Barañano, 2022 [19] (P4)	Neonate	n/a	yes	no	n/a
35	c.3115C>T	Gln1039Ter	Coil	Nonsense	Random 76:24	Symonds, 2017 [15] (P10)	2 M	none	yes	no	thin abnormally shaped CC and minimal cerebral atrophy
36	c.3145C>T	Arg1049Ter	Coil	Nonsense	n/a	Symonds, 2017 [15] (P2)	5–6 weeks	none	yes	no	cerebral volume loss
37	c.3241A>T	Ile1081Phe	Close to P2 head	Missense	n/a	Unreported	4 yr	none	yes	yes	mildly prominent lateral ventricles
38	c.3285+1G>C	p1095	Close to P2 head	Splice-site	n/a	Kruszka, 2019 [23] (P7)	not reported	n/a	yes	n/a	middle interhemispheric variant, HPE
39	c.3312C>A	Tyr1107Ter	Close to P2 head	Nonsense	n/a	Elwan, 2022 [20]	12 yr	normal	normal before SE	yes	normal
40	c.3326_3330delATGGCinsC	Asp1109Alafs*102	Close to P2 head	Frameshift	n/a	Symonds, 2017 [15] (P5)	6 M	none	yes	no	small cavum septum vergae
41	c.3549_3552dupGGCC	Ile1185glyfs*23	P2 head	Frameshift	Random in patient 1-Goldstein, 2015; n/a-P3- Barañano, 2022	Goldstein, 2015 [12] (P2); Barañano, 2022 [19] (P13)	17 M- P2- Goldstein 2015 [12]; 16 M- P13-Barañano 2022 [19]	n/a-Goldstein; P13-Barañano-limited	yes	n/a-Goldstein; P13Barababi-yes	P2 Goldstein-mild enlarged extra-axial spaces and slight thinning of CC

CC = corpus callosum; HPE = holoprosencephaly; P = patient; SE = status epilepticus; sz = seizure.

## Data Availability

All data supporting the conclusions of this manuscript are provided in the text and figures. Web Resources—The URLs for data presented include: OMIM: http://omim.org/ (accessed on 1 January 2023); ExAC: http://exac.broadinstitute.org/ (accessed on 5 December 2022); gnomAD: http://gnomad.broadinstitute.org (accessed on 5 December 2022); ClinVar: https://www.ncbi.nlm.nih.gov/clinvar/ (accessed on 5 December 2022).

## Supplementary Materials

The following supporting information can be downloaded at https://www.mdpi.com/article/10.3390/genes14040852/s1. Figure S1, Sanger sequencing confirms de novo SMC1A-DEE variants in three heterozygous female patients; Figure S2, random XCI patterns in the blood of the heterozygous SMC1A-DEE patient 1; Figure S3, random XCI patterns in the blood of the heterozygous SMC1A-DEE patient 2; Figure S4, skewed XCI patterns in the blood of the heterozygous SMC1A-DEE patient 3; Figure S5, the *SMC1A*-DEE variant escapes from skewed XCI in the blood of the heterozygous patient 3; Table S1, primers used in this study; Table S2, truncated protein sequence predicted from alternative transcripts of SMC1A in patient 1.
