# Supplementary material for: Phenotypes and Genotypes in Patients with SMC1A-Related Developmental and Epileptic Encephalopathy
Source: Genes (Basel). 2023 Mar 31;14(4):852. doi: 10.3390/genes14040852 (PMC10138066; doi:10.3390/genes14040852)
Supplement: Supplementary file 1 [file genes-14-00852-s001.zip › Figure S4 P3 XCI analysis.pptx]

## Slide 1
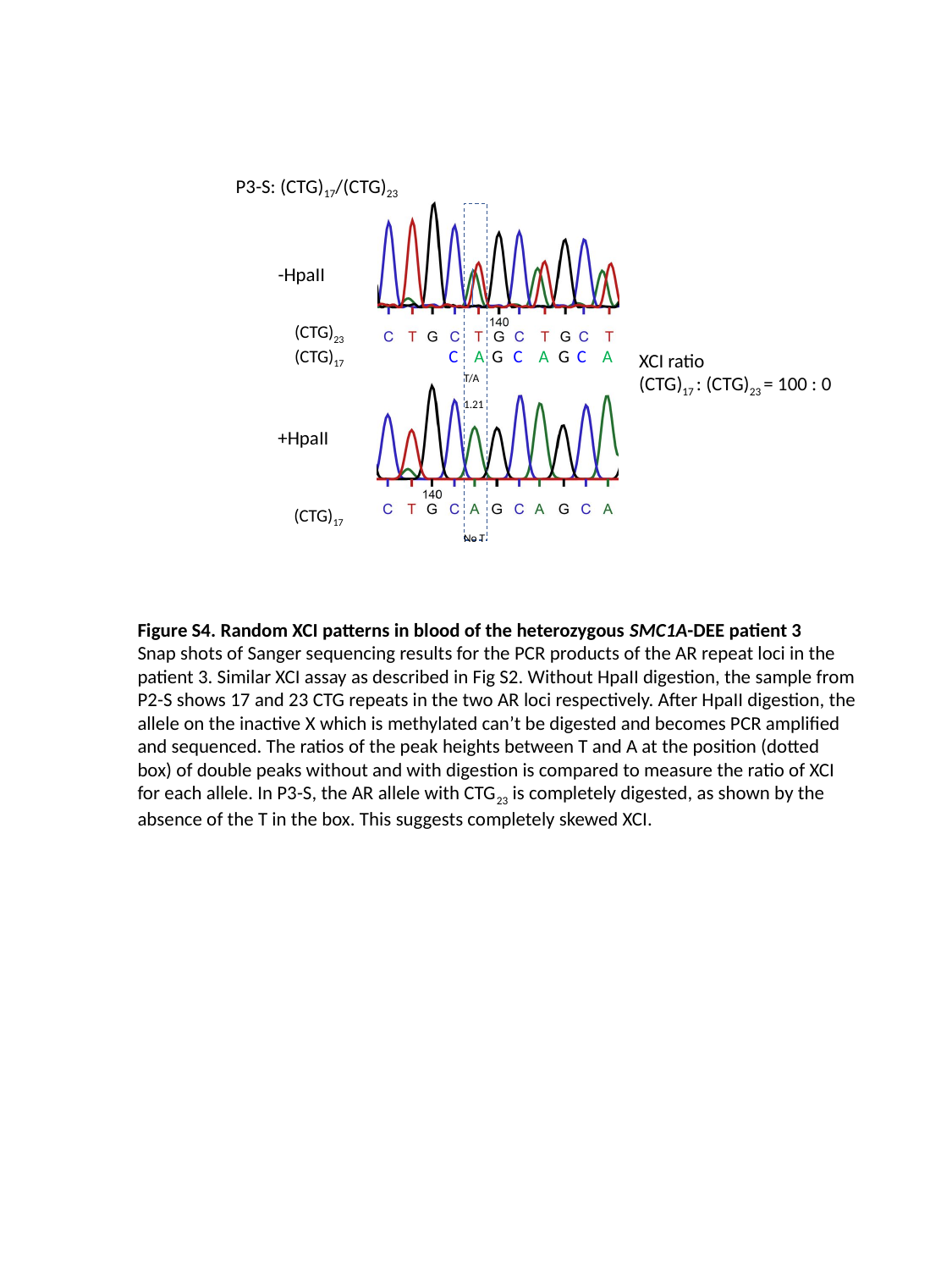

P3-S: (CTG)17/(CTG)23
-HpaII
(CTG)23
(CTG)17
C A G C A G C A
XCI ratio
(CTG)17 : (CTG)23 = 100 : 0
T/A
1.21
+HpaII
(CTG)17
No T
Figure S4. Random XCI patterns in blood of the heterozygous SMC1A-DEE patient 3
Snap shots of Sanger sequencing results for the PCR products of the AR repeat loci in the patient 3. Similar XCI assay as described in Fig S2. Without HpaII digestion, the sample from P2-S shows 17 and 23 CTG repeats in the two AR loci respectively. After HpaII digestion, the allele on the inactive X which is methylated can’t be digested and becomes PCR amplified and sequenced. The ratios of the peak heights between T and A at the position (dotted box) of double peaks without and with digestion is compared to measure the ratio of XCI for each allele. In P3-S, the AR allele with CTG23 is completely digested, as shown by the absence of the T in the box. This suggests completely skewed XCI.
