# Supplementary material for: Phenotypes and Genotypes in Patients with SMC1A-Related Developmental and Epileptic Encephalopathy
Source: Genes (Basel). 2023 Mar 31;14(4):852. doi: 10.3390/genes14040852 (PMC10138066; doi:10.3390/genes14040852)
Supplement: Supplementary file 1 [file genes-14-00852-s001.zip › Figure S5 P3 Escape in blood.pptx]

## Slide 1
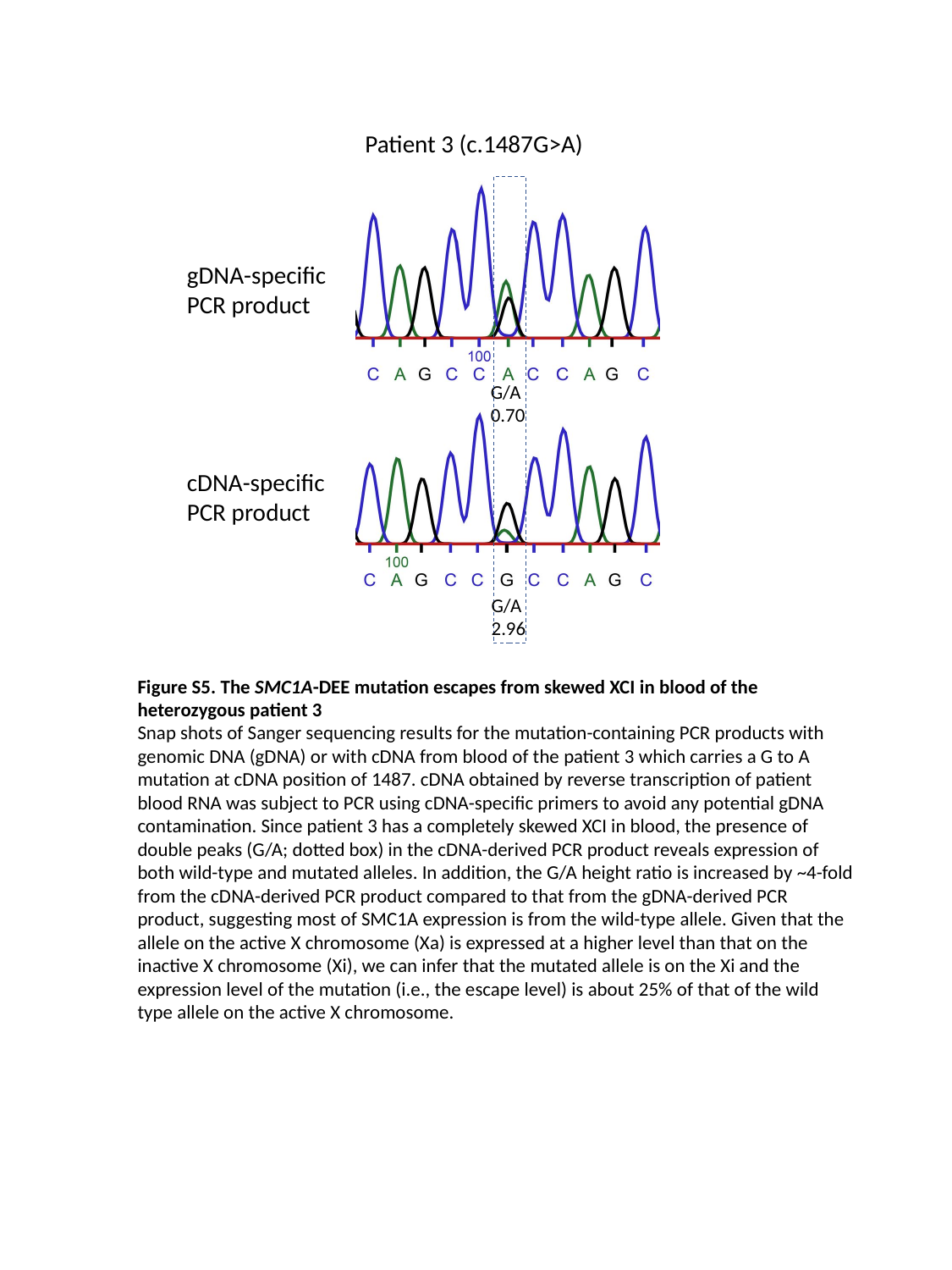

Patient 3 (c.1487G>A)
gDNA-specific PCR product
cDNA-specific PCR product
G/A
0.70
G/A
2.96
Figure S5. The SMC1A-DEE mutation escapes from skewed XCI in blood of the heterozygous patient 3
Snap shots of Sanger sequencing results for the mutation-containing PCR products with genomic DNA (gDNA) or with cDNA from blood of the patient 3 which carries a G to A mutation at cDNA position of 1487. cDNA obtained by reverse transcription of patient blood RNA was subject to PCR using cDNA-specific primers to avoid any potential gDNA contamination. Since patient 3 has a completely skewed XCI in blood, the presence of double peaks (G/A; dotted box) in the cDNA-derived PCR product reveals expression of both wild-type and mutated alleles. In addition, the G/A height ratio is increased by ~4-fold from the cDNA-derived PCR product compared to that from the gDNA-derived PCR product, suggesting most of SMC1A expression is from the wild-type allele. Given that the allele on the active X chromosome (Xa) is expressed at a higher level than that on the inactive X chromosome (Xi), we can infer that the mutated allele is on the Xi and the expression level of the mutation (i.e., the escape level) is about 25% of that of the wild type allele on the active X chromosome.
