# Supplementary material for: Phenotypes and Genotypes in Patients with SMC1A-Related Developmental and Epileptic Encephalopathy
Source: Genes (Basel). 2023 Mar 31;14(4):852. doi: 10.3390/genes14040852 (PMC10138066; doi:10.3390/genes14040852)
Supplement: Supplementary file 1 [file genes-14-00852-s001.zip › Figure S1 Confirmation of SMC1A de novo mutations.pptx]

## Slide 1
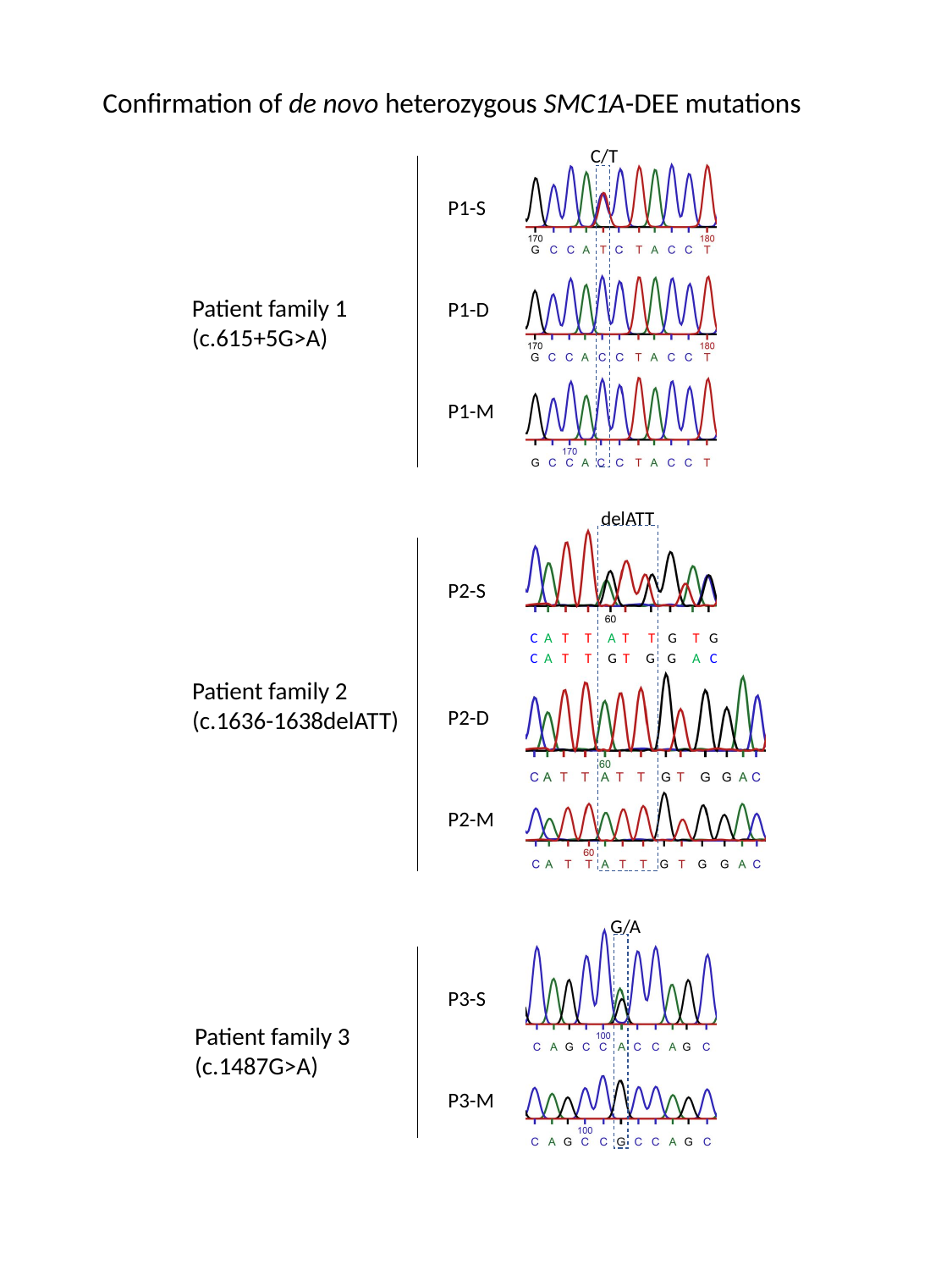

Confirmation of de novo heterozygous SMC1A-DEE mutations
C/T
P1-S
P1-D
P1-M
Patient family 1
(c.615+5G>A)
delATT
P2-S
P2-D
P2-M
C A T T A T T G T G
C A T T G T G G A C
Patient family 2
(c.1636-1638delATT)
G/A
P3-S
P3-M
Patient family 3
(c.1487G>A)

## Slide 2
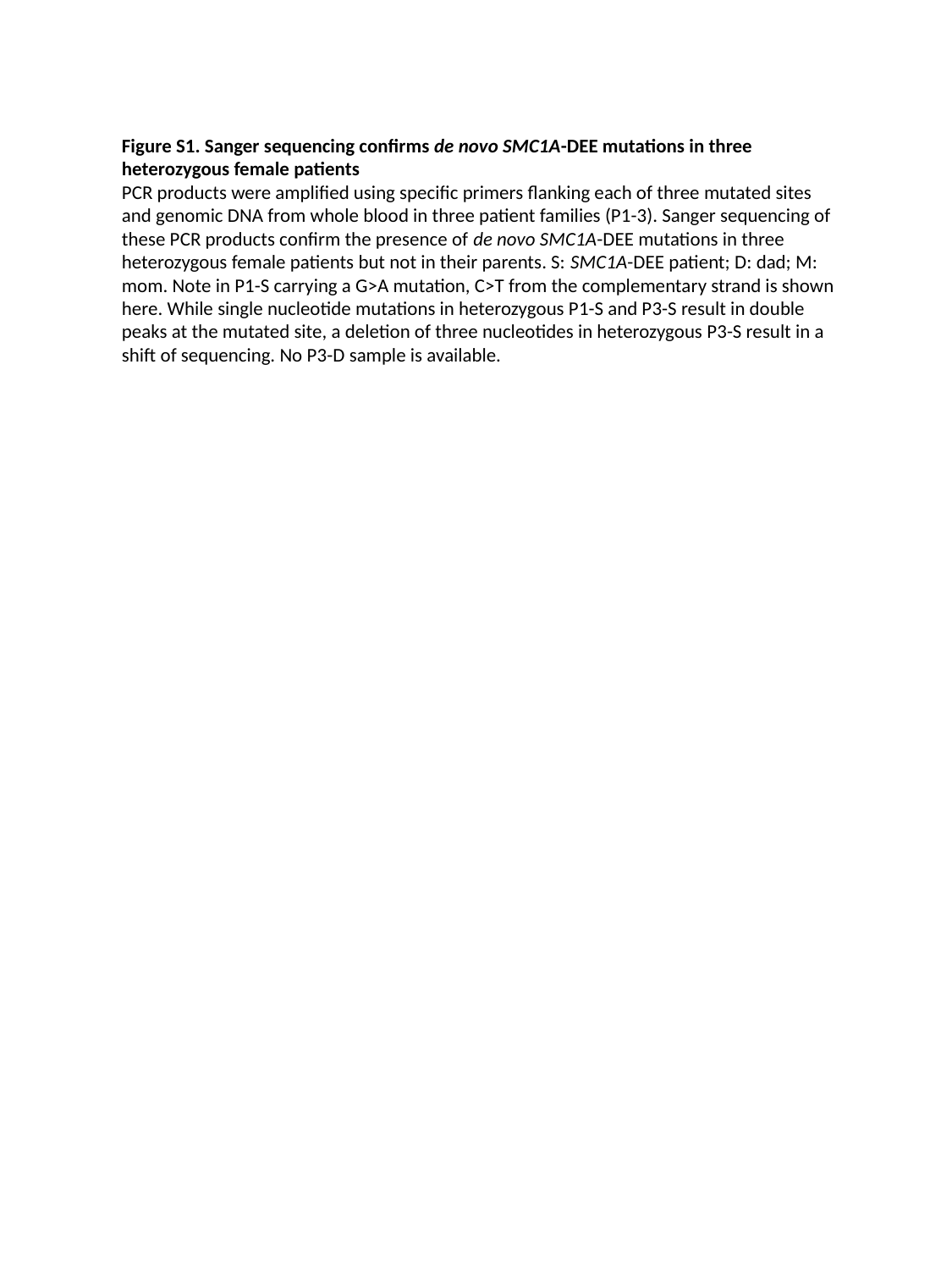

Figure S1. Sanger sequencing confirms de novo SMC1A-DEE mutations in three heterozygous female patients
PCR products were amplified using specific primers flanking each of three mutated sites and genomic DNA from whole blood in three patient families (P1-3). Sanger sequencing of these PCR products confirm the presence of de novo SMC1A-DEE mutations in three heterozygous female patients but not in their parents. S: SMC1A-DEE patient; D: dad; M: mom. Note in P1-S carrying a G>A mutation, C>T from the complementary strand is shown here. While single nucleotide mutations in heterozygous P1-S and P3-S result in double peaks at the mutated site, a deletion of three nucleotides in heterozygous P3-S result in a shift of sequencing. No P3-D sample is available.
