# Supplementary material for: Phenotypes and Genotypes in Patients with SMC1A-Related Developmental and Epileptic Encephalopathy
Source: Genes (Basel). 2023 Mar 31;14(4):852. doi: 10.3390/genes14040852 (PMC10138066; doi:10.3390/genes14040852)
Supplement: Supplementary file 1 [file genes-14-00852-s001.zip › Figure S2 P1 XCI analysis.pptx]

## Slide 1
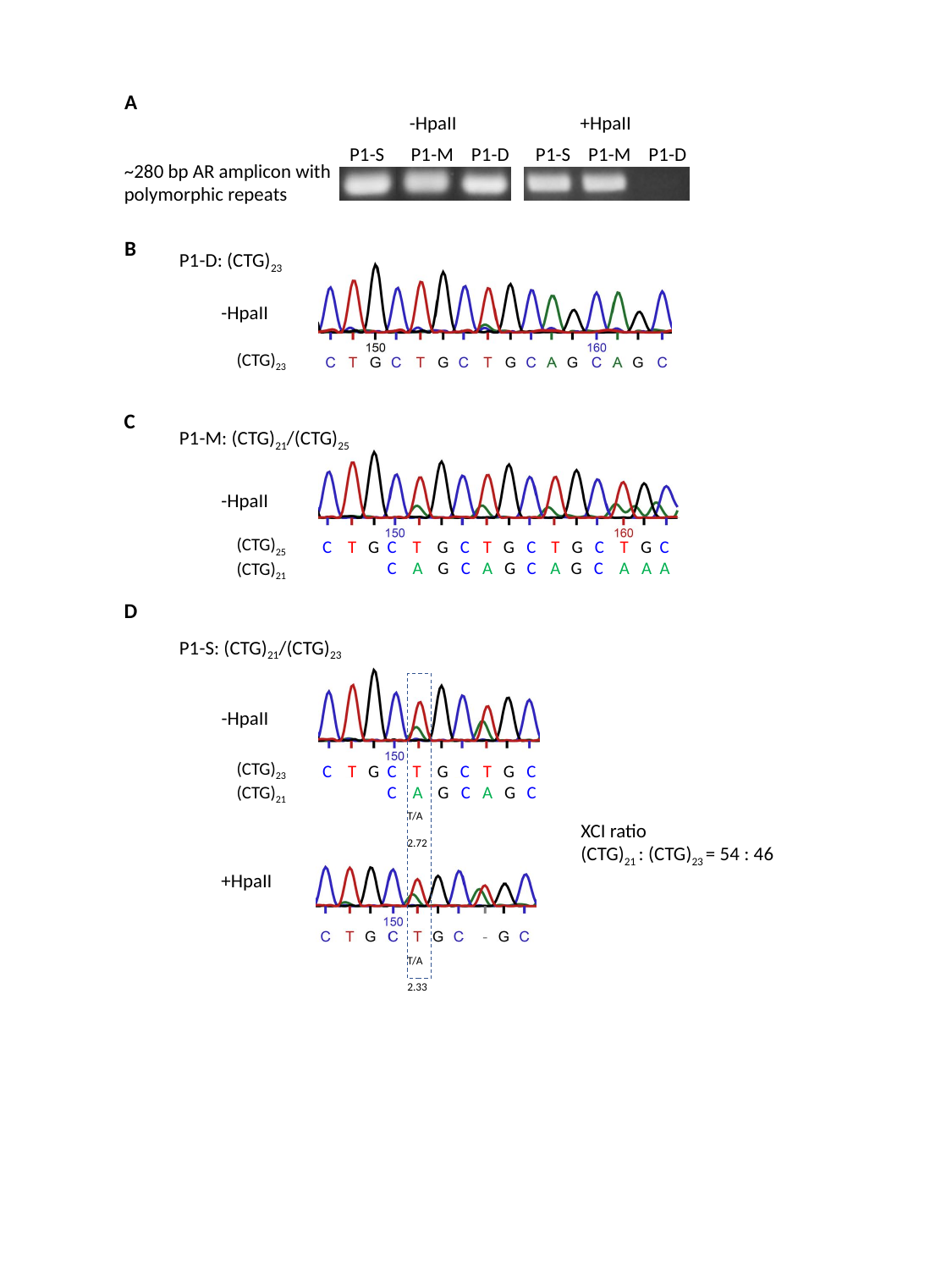

A
-HpaII +HpaII
P1-S P1-M P1-D P1-S P1-M P1-D
~280 bp AR amplicon with polymorphic repeats
B
P1-D: (CTG)23
-HpaII
(CTG)23
C
P1-M: (CTG)21/(CTG)25
-HpaII
(CTG)25
(CTG)21
C T G C T G C T G C T G C T G C
C A G C A G C A G C A A A
D
P1-S: (CTG)21/(CTG)23
-HpaII
(CTG)23
(CTG)21
C T G C T G C T G C
C A G C A G C
T/A
2.72
XCI ratio
(CTG)21 : (CTG)23 = 54 : 46
+HpaII
T/A
2.33

## Slide 2
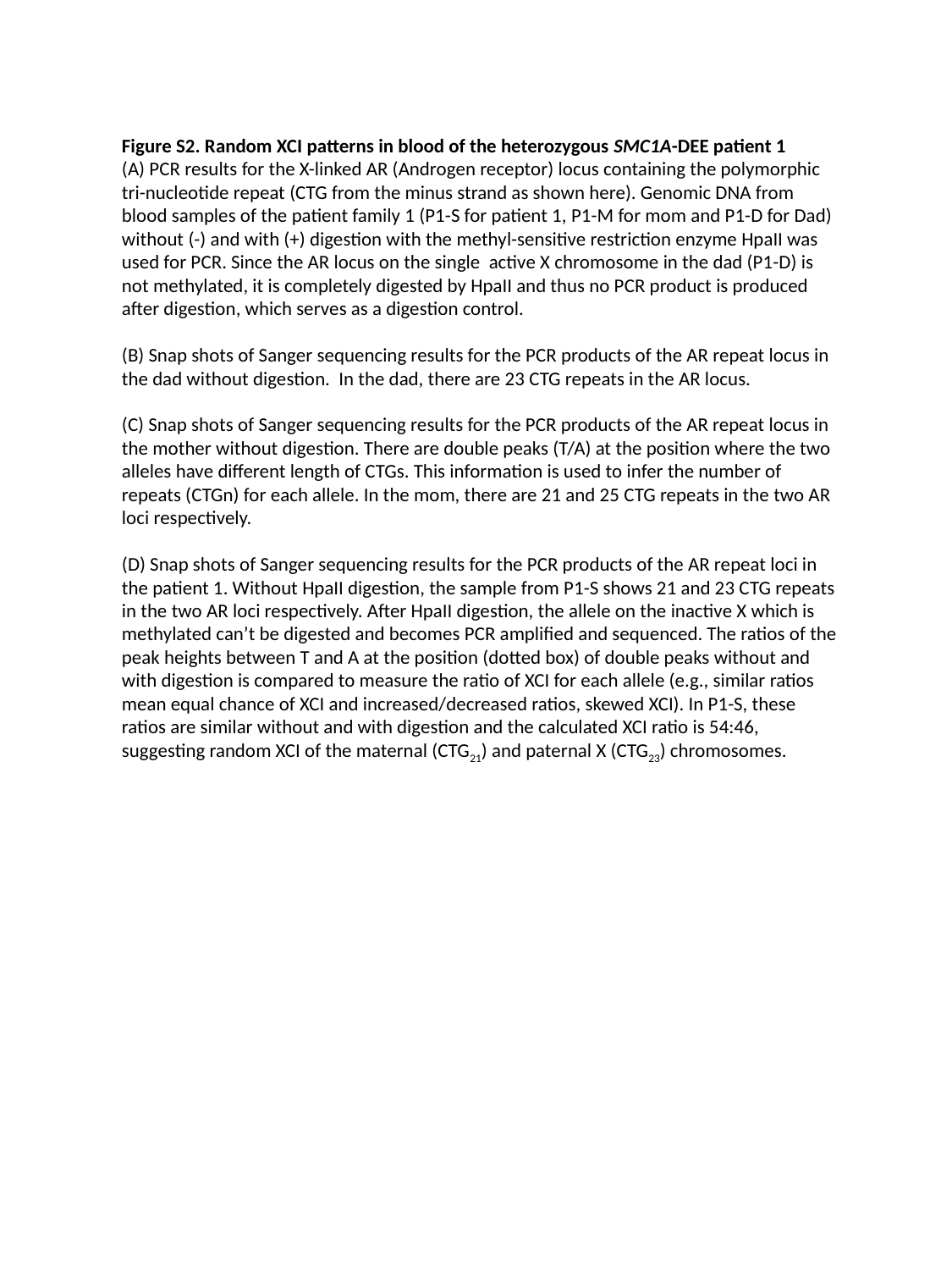

Figure S2. Random XCI patterns in blood of the heterozygous SMC1A-DEE patient 1
(A) PCR results for the X-linked AR (Androgen receptor) locus containing the polymorphic tri-nucleotide repeat (CTG from the minus strand as shown here). Genomic DNA from blood samples of the patient family 1 (P1-S for patient 1, P1-M for mom and P1-D for Dad) without (-) and with (+) digestion with the methyl-sensitive restriction enzyme HpaII was used for PCR. Since the AR locus on the single active X chromosome in the dad (P1-D) is not methylated, it is completely digested by HpaII and thus no PCR product is produced after digestion, which serves as a digestion control.
(B) Snap shots of Sanger sequencing results for the PCR products of the AR repeat locus in the dad without digestion. In the dad, there are 23 CTG repeats in the AR locus.
(C) Snap shots of Sanger sequencing results for the PCR products of the AR repeat locus in the mother without digestion. There are double peaks (T/A) at the position where the two alleles have different length of CTGs. This information is used to infer the number of repeats (CTGn) for each allele. In the mom, there are 21 and 25 CTG repeats in the two AR loci respectively.
(D) Snap shots of Sanger sequencing results for the PCR products of the AR repeat loci in the patient 1. Without HpaII digestion, the sample from P1-S shows 21 and 23 CTG repeats in the two AR loci respectively. After HpaII digestion, the allele on the inactive X which is methylated can’t be digested and becomes PCR amplified and sequenced. The ratios of the peak heights between T and A at the position (dotted box) of double peaks without and with digestion is compared to measure the ratio of XCI for each allele (e.g., similar ratios mean equal chance of XCI and increased/decreased ratios, skewed XCI). In P1-S, these ratios are similar without and with digestion and the calculated XCI ratio is 54:46, suggesting random XCI of the maternal (CTG21) and paternal X (CTG23) chromosomes.
