# Supplementary material for: Phenotypes and Genotypes in Patients with SMC1A-Related Developmental and Epileptic Encephalopathy
Source: Genes (Basel). 2023 Mar 31;14(4):852. doi: 10.3390/genes14040852 (PMC10138066; doi:10.3390/genes14040852)
Supplement: Supplementary file 1 [file genes-14-00852-s001.zip › Figure S3 P2 XCI analysis.pptx]

## Slide 1
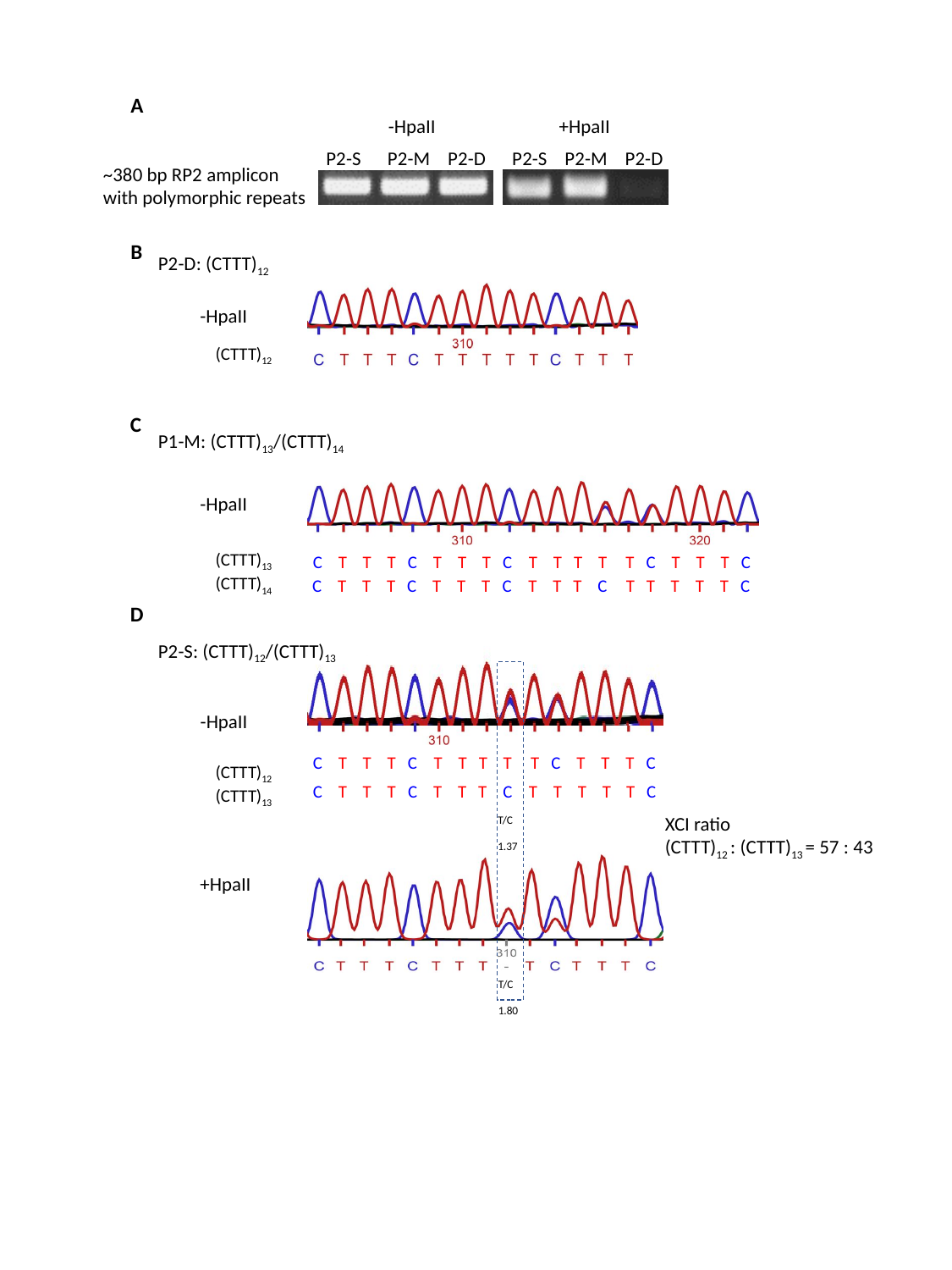

A
-HpaII +HpaII
P2-S P2-M P2-D P2-S P2-M P2-D
~380 bp RP2 amplicon with polymorphic repeats
B
P2-D: (CTTT)12
-HpaII
(CTTT)12
C
P1-M: (CTTT)13/(CTTT)14
-HpaII
(CTTT)13
(CTTT)14
C T T T C T T T C T T T T T C T T T C
C T T T C T T T C T T T C T T T T T C
D
P2-S: (CTTT)12/(CTTT)13
-HpaII
C T T T C T T T T T C T T T C
(CTTT)12
(CTTT)13
C T T T C T T T C T T T T T C
T/C
1.37
XCI ratio
(CTTT)12 : (CTTT)13 = 57 : 43
+HpaII
T/C
1.80

## Slide 2
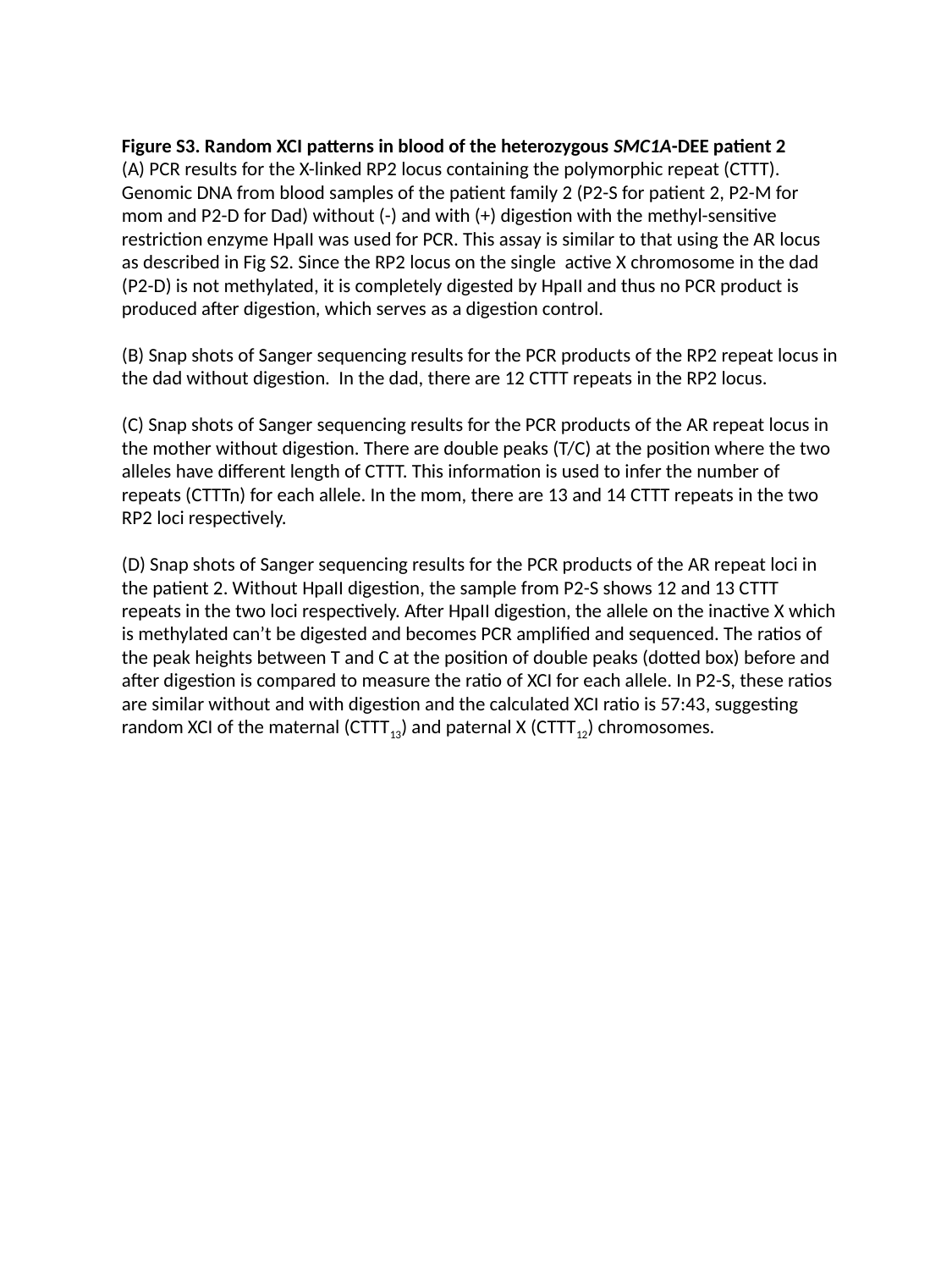

Figure S3. Random XCI patterns in blood of the heterozygous SMC1A-DEE patient 2
(A) PCR results for the X-linked RP2 locus containing the polymorphic repeat (CTTT). Genomic DNA from blood samples of the patient family 2 (P2-S for patient 2, P2-M for mom and P2-D for Dad) without (-) and with (+) digestion with the methyl-sensitive restriction enzyme HpaII was used for PCR. This assay is similar to that using the AR locus as described in Fig S2. Since the RP2 locus on the single active X chromosome in the dad (P2-D) is not methylated, it is completely digested by HpaII and thus no PCR product is produced after digestion, which serves as a digestion control.
(B) Snap shots of Sanger sequencing results for the PCR products of the RP2 repeat locus in the dad without digestion. In the dad, there are 12 CTTT repeats in the RP2 locus.
(C) Snap shots of Sanger sequencing results for the PCR products of the AR repeat locus in the mother without digestion. There are double peaks (T/C) at the position where the two alleles have different length of CTTT. This information is used to infer the number of repeats (CTTTn) for each allele. In the mom, there are 13 and 14 CTTT repeats in the two RP2 loci respectively.
(D) Snap shots of Sanger sequencing results for the PCR products of the AR repeat loci in the patient 2. Without HpaII digestion, the sample from P2-S shows 12 and 13 CTTT repeats in the two loci respectively. After HpaII digestion, the allele on the inactive X which is methylated can’t be digested and becomes PCR amplified and sequenced. The ratios of the peak heights between T and C at the position of double peaks (dotted box) before and after digestion is compared to measure the ratio of XCI for each allele. In P2-S, these ratios are similar without and with digestion and the calculated XCI ratio is 57:43, suggesting random XCI of the maternal (CTTT13) and paternal X (CTTT12) chromosomes.
